# Tingles, Tetany, and Electrolyte Derangements

**DOI:** 10.7759/cureus.7854

**Published:** 2020-04-27

**Authors:** Amardeep Singh, Ramandeep Kaur, Bhagwan Dass, Abutaleb Ejaz

**Affiliations:** 1 Nephrology, University of Florida Health, Gainesville, USA; 2 Miscellaneous, Virginia Commonwealth University, Richmond, USA

**Keywords:** hypomagnesemia, hypocalcemia, hypokalemia, mechanisms, electrolyte disturbances, tetany, paresthesia, chronic kidney disease, genetic syndromes, general internal medicine

## Abstract

We report a patient who presented with anxiety, hyperventilation, perioral paresthesia, and tingling in the fingers associated with hypomagnesemia, hypocalcemia, and hypokalemia. We discuss the possible mechanistic basis for sequence of events that may have led to this presentation.

## Introduction

Hypomagnesemia is defined as serum magnesium level <1.5 mEq/L and can be seen in ~12% of hospitalized patients. It is typically associated with volume expansion, chronic diarrhea, diuretic and antibiotic use, and malnutrition [1]. Hypomagnesemia can cause life-threatening cardiac arrhythmias through its influence on potassium and calcium homeostasis. We discuss the interconnectedness of magnesium, potassium, and calcium in this case report.

## Case presentation

A 47-year-old female presented to the ER with severe anxiety, palpitations, and hyperventilation associated with perioral tingling and numbness of the fingers that started several hours previously. She had similar episodes two years ago without elucidations of the etiology of her symptoms. She was not on any medication; social history was negative for alcohol or illicit drug use.

On physical examination, blood pressure was 131/83 mmHg, heart rate 122 beats per minute, respiratory rate 16 per minute, temperature 36.7°C, and oxygen saturation 98%. The patient was alert, oriented, and appeared much younger than the stated age. Chvostek and Trousseau signs were negative. She, however, continued to complain of perioral paresthesia and twitching (which was not visually evident). Cardiovascular, respiratory, gastrointestinal, and neurological examinations were unremarkable. Laboratory findings included, serum sodium 140 mEq/L (normal 135-145 mEq/L), potassium 2.7 mEq/L (normal 3.5-5.0 mEq/L), chloride 105 mEq/L (normal 98-106 mEq/L), bicarbonate 13 mEq/L (normal 23-28 mEq/L), blood urea nitrogen (BUN) 29 mg/dL (normal 7-20 mg/dL), creatinine 2.5 mg/dL (normal 0.7-1.3 mg/dL), calcium 7.8 mg/dL (normal 9-10.5 mg/dL), magnesium 0.9 mg/dL (normal 1.5-2.4 mg/dL), phosphorus 2.3 mg/dL (normal 3-4.5 mg/dL), ionized calcium 1.01 mmol/L (normal 1.2-1.4 mmol/L), parathyroid hormone (PTH) 998 pg/mL (normal 10-65 pg/mL), anion gap 12 and delta gap -1 (compatible with high anion gap acidosis). Urine pH was 7.4, random urine calcium and creatinine were 3 and 29.9 mg/dL, respectively. Calculated fractional excretion of calcium was 0.03%. Urine drug screen for amphetamines, methamphetamines, benzodiazepines, barbiturates, marijuana, cocaine, phencyclidine (PCP), methadone, and opioids were negative. A 24-h urine calcium was 47 mg/24 h (normal 50-100 mg/24 h). Plasma renin activity 1.4 ng/mL/h (normal 2.9-24 ng/mL/h), supine aldosterone 2.3 ng/dL (normal 3-35 ng/dL), 25-OH vitamin D 28 ng/mL (normal 20-50 ng/mL), and 1,25-OH vitamin D 51 ng/mL (normal 25-65 ng/mL). Arterial blood gas was not available.

Electrocardiogram revealed sinus tachycardia with a PR interval of 13 ms, QRS of 87 ms, and QT 282 ms. Fluid resuscitation was initiated with 2 L of normal saline. Additives included magnesium sulfate, followed by potassium chloride and calcium chloride. Serum potassium improved to 3.4 mEq/L, magnesium to 2.2 mEq/L, and calcium to 1.25 mmol/L and the patient had an output of 1.2 L. In the next four days her electrolytes returned to normal levels, BUN and serum creatinine improved to 10 and 1.1 mg/dL, respectively. She was discharged from the hospital with instruction for outpatient follow-up within seven days. She did not show-up for her clinic appointment and was lost to follow-up.

## Discussion

The patient’s symptoms improved with correction of hypomagnesemia and hypocalcemia. The pivotal question is the etiology and the mechanisms involved here. A brief discussion of calcium, magnesium, and potassium homeostasis is in order. The daily dietary calcium load (~1000 mg) is predominantly excreted in feces (90%) and only 10% by the kidneys. PTH and 1,25-dihydroxyvitamin D are the principal players in normal calcium homeostasis. A decrease in the serum ionized calcium concentration and a decrease in serum levels of 1,25(OH)2-D trigger PTH secretion leading to increased conversion of 25-hydroxy (OH) vitamin D to the potent 1,25-dihydroxyvitamin D by the kidneys and stimulation of increased intestinal absorption of calcium [2].

In the kidney, approximately 70% of filtered calcium is reabsorbed in the proximal tubule through a paracellular pathway predominantly through solvent drag [3]. Some 20% of the filtered calcium is reabsorbed by the thick ascending limb by paracellular pathway (tight junctions composed mostly of paracellin-1) driven by lumen-positive voltage created and maintained by NKCC2, Na+/K+-ATPase, and chloride channel ClC-Kb [4]. Renal outer medullary potassium channel (ROMK) mediates K+ ion recycling across apical plasma membranes to the luminal fluid of the thick ascending limb and is responsible for generating the lumen-positive voltage that serves as a driving force for the cation-selective paracellular movement of Ca2+, Mg2+, and of additional Na+ absorption by the TAL. ROMK is regulated by calcium sensing receptors [5]. Extracellular Ca2+ inhibits both NKCC2 and ROMK, decreasing salt reabsorption. Only 8% of filtered calcium is reabsorbed by the distal tubule via active transcellular pathways.

Filtered magnesium is reabsorbed in the proximal tubule (~25%) and thick ascending limb (~60%) via paracellular pathways involving transmembrane proteins paracellin-1 and claudin-19 and driven by potential gradients. Magnesium is also reabsorbed in the distal tubules (~5%) via transcellular pathways that involve transient receptor potential (TRPM6) [6]. Magnesium deficiency is known to exacerbate urinary potassium loss by increasing distal potassium secretion. A decrease in intracellular magnesium releases the magnesium-mediated inhibition of ROMK channels and increases potassium secretion. This process requires concomitant increase in distal sodium delivery or elevated aldosterone levels.

In our patient serum potassium, magnesium, and calcium improved with supplements. Low fractional excretion of calcium was an appropriate response by the kidneys to conserve calcium in the presence of hypocalcemia, possibly via increased distal tubule active transcellular pathways. The sequence of events that resulted in the patient’s symptoms is uncertain, but may have been precipitated by hypomagnesemia, followed by hypokalemia and hypocalcemia manifesting as perioral tingling and numbness of the fingers via mechanisms discussed above. Hypocalcemia was the presumed trigger for increased PTH secretion. The etiology of hypomagnesemia, whether acquired (poor dietary intake, gastrointestinal absorption issues, or self-induced) versus genetic factors remains unknown. 

## Conclusions

Hypomagnesemia can cause adverse clinical events through its effect on downstream systems that control potassium and calcium as shown in this case. The serum magnesium level that can result in adverse events is unknown as there is no linear correlation with total body magnesium and serum magnesium levels. Correction of magnesium significantly improved potassium and magnesium levels and symptoms in this patient. Our case offers a glimpse of the complex interconnectedness of ion transport systems in the human body. 
